# Improvement of balance in young adults by a sound component at 100 Hz in music

**DOI:** 10.1038/s41598-018-35244-3

**Published:** 2018-11-15

**Authors:** Huadong Xu, Nobutaka Ohgami, Tingchao He, Kazunori Hashimoto, Akira Tazaki, Kyoko Ohgami, Kozue Takeda, Masashi Kato

**Affiliations:** 1 Department of Occupational and Environmental Health, Nagoya University Graduate School of Medicine, Nagoya, Japan; 2 Department of Biomedical Sciences, College of Life and Health Sciences, Chubu University, 1200 Matsumoto, Kasugai, Aichi 487-8501 Japan; 3 Voluntary Body for International Health Care in Universities, Nagoya, Japan

## Abstract

About 80% of young people use personal listening devices (PLDs) including MP3 players to listen to music, which consists of sound components with various frequencies. Previous studies showed that exposure to noise of high intensities affected balance in humans. However, there is no information about a frequency-dependent effect of sound components in music from a PLD on balance in young people. In this study, we determined the associations between sound component levels (dB) at 100, 1000 and 4000 Hz in music from a portable listening device (PLD) and balance objectively determined by posturography in young adults (n = 110). We divided the subjects into two groups (low and high exposure groups) based on cut-off values of sound component levels at each frequency using receiver operating characteristic (ROC) curves. Balance in the high exposure group (≥46.6 dB) at 100 Hz was significantly better than that in low exposure group in logistic regression models adjusted for sex, BMI, smoking status and alcohol intake, while there were no significant associations at 1000 and 4000 Hz. Thus, this study demonstrated for the first time that the sound component at 100 Hz with more than 46.6 dB in music improved balance in young adults.

## Introduction

Personal listening devices (PLDs) including MP3 players, cellphones and other portal music players have become increasingly popular, especially among adolescents and young adults^1,2^. A previous study showed that the proportions of undergraduate, post-graduate and community college students in USA who owned PLDs had reached 84%, 86% and 72%, respectively^3^. Thus, about 80% of young people use PLDs in their daily life.

Music output from a PLD consists of sound components with various frequencies (Hz). Many epidemiological studies have shown associations of exposure to sound levels (dB) from a PLD with noise-induced hearing loss^3–5^ and with other health effects including cardiac autonomic modulation^6^. Thus, the sound levels from a PLD have been mainly used to evaluate the health risks associated with a PLD. Exposure to sound components with different frequencies in occupational noise has also been shown to be associated with some health risks other than hearing loss. A previous study showed that occupational exposure to a sound component at 4000 Hz with sound levels above 70 dB is associated with hypertension as well as noise-induced hearing loss in humans^7,8^. Exposure to noise with frequencies of 10–250 Hz has been shown to be associated with annoyance^9^. Thus, it is possible that sound components with specific frequencies in music affect not only hearing levels but also physical functions in humans. However, there is very limited information about associations between sound components at different frequencies in music output from a PLD and physiological functions other than hearing.

Balance is a fundamental function in daily life. Loss of balance has been shown to affect postural stability that can result in an increased incidence of falls in adults^10^. Posturography has been used to determine balance in humans by measurement of body sway^11^. In posturography, track length and surface area of the center of pressure with eyes open and eyes closed are measured to assess balance^12^ and larger values of track length and surface area indicate worse performance of balance^13^. Previous studies showed that subjects exposed to noise of high intensities had significantly larger postural sway^14,15^. Thus, it is possible that exposure to noise affects balance. However, the associations of sound components at different frequencies in music output from a PLD with balance are not clear.

In this epidemiological study, we measured sound components at 100, 1000 and 4000 Hz in music output from a PLD by using a noise meter equipped with fast fourier transform (FFT) and we objectively determined balance by posturography in young adults (n = 110) in order to determine the associations between sound levels of sound components at different frequencies and balance.

## Results

### Basic characteristics of the subjects

**Figure 1:**
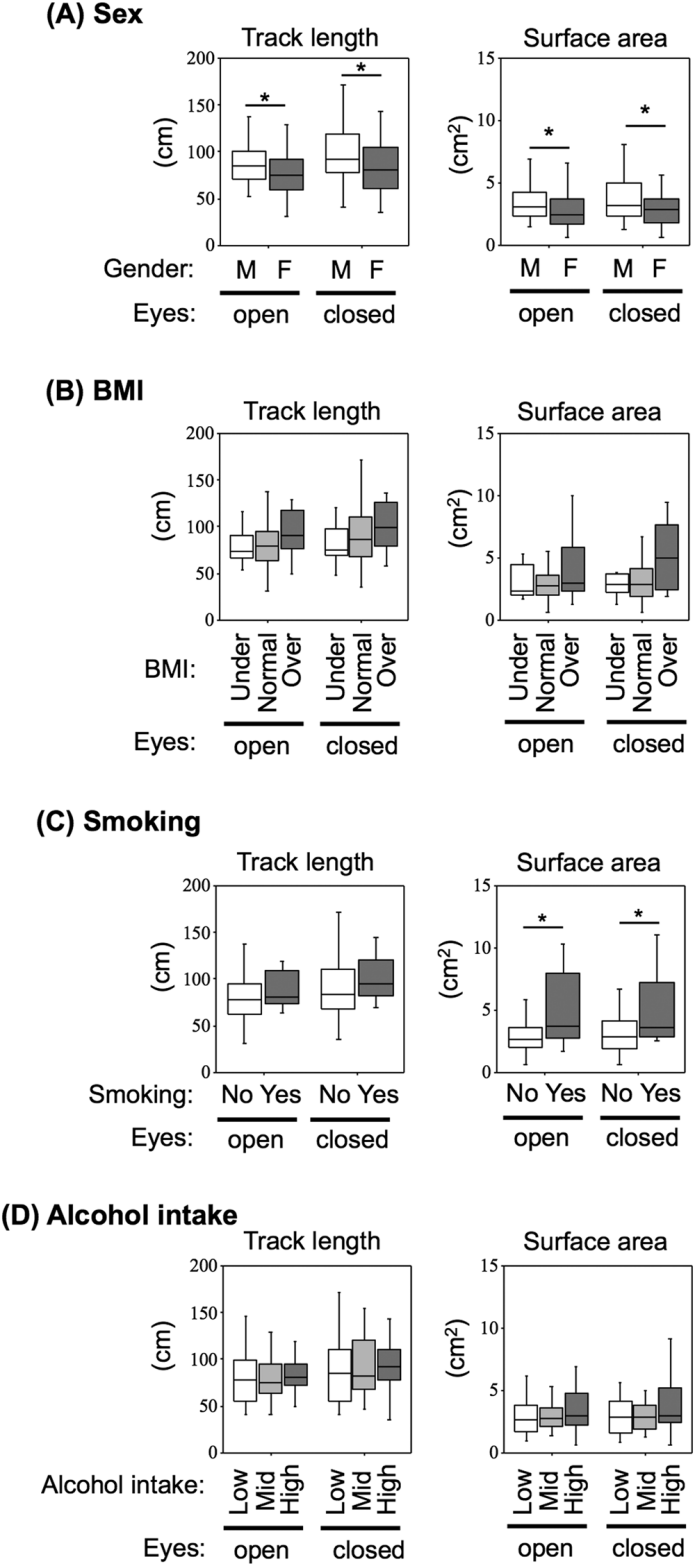
Associations of balance with confounding factors including sex, BMI, smoking and alcohol. Track lengths (left box plots) and surface areas (right box plots) recorded with eyes open (open) and eyes closed (closed) in the male group (M; n = 52) and female group (F; n = 58) (**A**), in the underweight group (Under; n = 16), normal weight group (Normal; n = 83) and overweight weight group (Over; n = 11) (**B**), in the non-smokers (No; n = 96) and smokers (Yes; n = 14), in the low alcohol consumption group (Low; n = 35), middle alcohol consumption group (Mid; n = 39) and high alcohol consumption group (High; n = 36) (**D**) are displayed. Cut-off values of BMI (**B**) and alcohol intake (**D**) are shown in Table 1. Significant differences (*p < 0.05) were analyzed by the Mann-Whitney *U* test (**A**,**C**) and Kruskal-Wallis H test (**C**,**D**).

The basic characteristics of the subjects including age, sex, BMI, smoking status and alcohol consumption are shown in Table 1. The subjects included 52 females and 58 males with an average age and average BMI of 20.4 ± 1.0 years and 21.4 ± 3.3 kg/m^2^, respectively. The values of track length and surface area with eyes open (88.65 ± 27.13 cm and 4.00 ± 2.87 cm^2^, respectively) and with eyes closed (100.80 ± 38.77 cm and 4.06 ± 2.50 cm^2^, respectively) in males were significantly higher than those with eyes open (76.34 ± 23.61 cm, 2.93 ± 1.81 cm^2^) and with eyes closed (85.81 ± 32.82 cm and 3.15 ± 2.08 cm^2^, respectively) in females (p < 0.05) (Fig. 1A). The subjects were divided into three groups based on BMI according to the WHO categories. There was no significant difference in any of the scores of posturography among the three BMI groups (Fig. 1B). Smokers (n = 14) had significantly larger surface areas with both eyes open (4.88 ± 3.59 cm^2^) and eyes closed (4.84 ± 2.76 cm^2^) than those of eyes open (3.23 ± 2.15 cm^2^) and eyes closed (3.40 ± 2.21 cm^2^) in non-smokers (n = 96) (p < 0.05) (Fig. 1C, right graph). The subjects were also classified into three groups based on tertiles of alcohol consumption per week to examine the potential relationship between alcohol consumption and balance. There was no significant difference among the three groups (Fig. 1D).

**Table 1:** General Information on the Study Participants (n = 110).

Characteristics	Variables	Participants (n)	Percentage (%)
Age (years)	20.4 ± 1.0	110	100
Sex	Male	52	47.3
	Female	58	52.7
BMI (kg/m^2^)	Underweight (<18.5)	16	14.5
	Normal weight (18.6–24.9)	83	75.5
	Overweight (≥25)	11	10.0
Smoking	No	96	87.3
	Yes	14	12.7
Alcohol intake (mL/week)	Low (<5.3)	35	31.8
	Mid (5.4–19.3)	39	35.5
	High (≥19.4)	36	32.7

### Comparison of the scores of posturography in the low and high exposure groups at 100, 1000 and 4000 Hz output from a PLD

**Figure 2:**
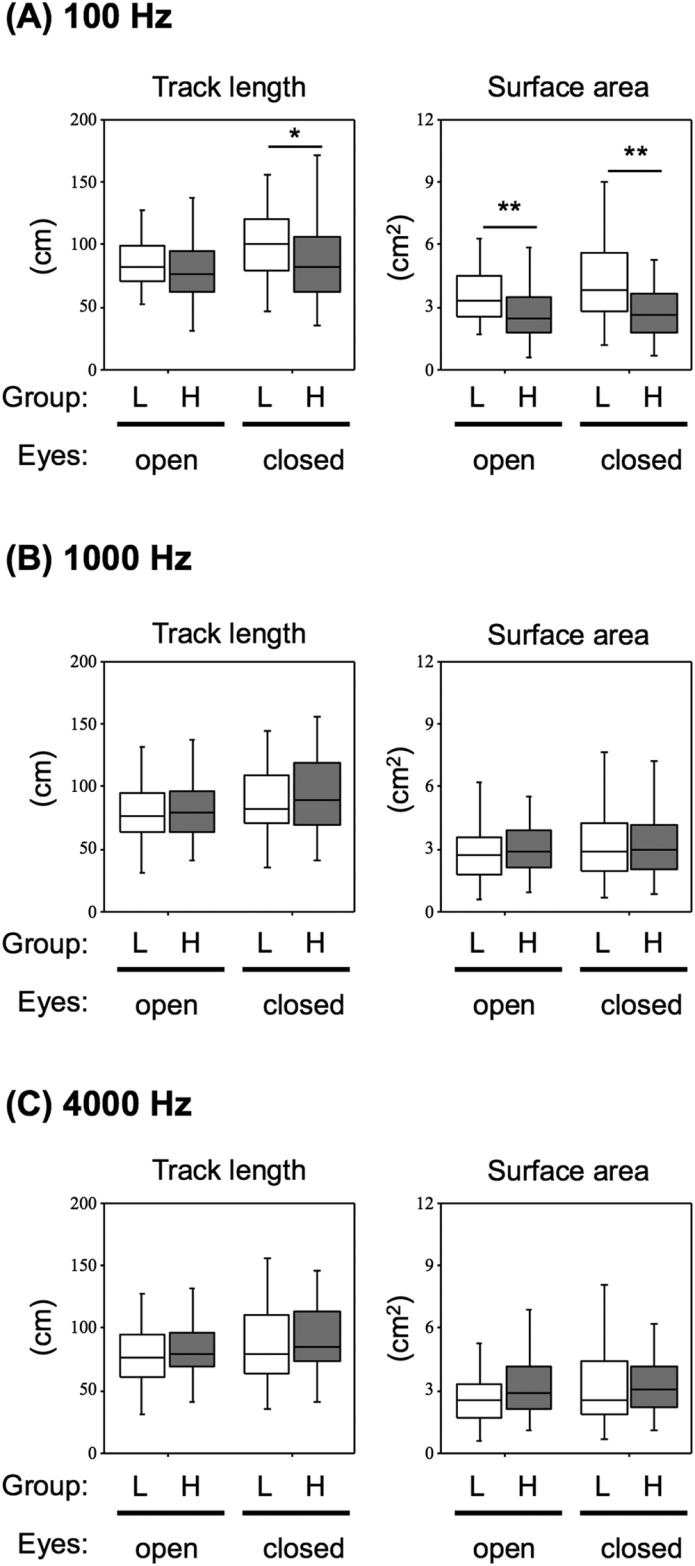
Associations of balance with sound levels at different frequencies output from the PLD. Track lengths (left box plots) and surface areas (right box plots) recorded with eyes open (open) and eyes closed (closed) in the low exposure group (L) and high exposure group (H) at 100 Hz (**A**), 1000 Hz (**B**) and 4000 Hz (**C**) of sound output from the PLD are presented. Cut-off values of sound levels at different frequencies are shown in Table 2. Significant differences (*p < 0.05, **p < 0.01) were analyzed by the Mann-Whitney *U* test.

As shown in Table 2, sound levels at 100, 1000 and 4000 Hz output from a PLD were 48.4 ± 4.0, 34.2 ± 10.3 and 40.4 ± 12.7 dB, respectively. Background sound levels at 100, 1000 and 4000 Hz were 40.2, 17.8 and 15.6 dB, respectively. To determine the associations of sound levels at different frequencies with postural stability, we categorized the subjects into two groups (low and high exposure groups) based on cutoff values of sound levels at each frequency using ROC curves. We compared the scores of track length and surface area in posturography between the low and high exposure groups at 100, 1000 and 4000 Hz (Fig. 2). The results showed that surface areas with eyes open (3.24 ± 2.62 cm^2^) and eye closed (3.20 ± 2.25 cm^2^) in the high exposure group at 100 Hz were significantly smaller than those with eyes open (3.88 ± 1.86 cm^2^) and eye closed (4.44 ± 2.28 cm^2^) in the low exposure group (Fig. 2A, right graph). Track length with eyes closed (88.56 ± 35.66 cm) in the high exposure group at 100 Hz was also less than that (102.59 ± 36.60 cm) in the low exposure group (*p* = 0.038) (Fig. 2A, left graph). Comparisons between two groups categorized at 1000 and 4000 Hz showed that there were no significant differences between the low and high exposure groups (Fig. 2B,C).

**Table 2:** Sound levels and background at different frequencies output from a PLD (n = 110).

Frequencies	Sound levels (dB)				Participants (n)
	Mean ± SD	Background	Maximum	Cut-off value*	
100 Hz	48.4 ± 4.0	40.2	68.1	Low (<46.6)	34
				High (≥46.6)	76
1000 Hz	34.2 ± 10.3	17.8	74.9	Low (<33.0)	56
				High (≥33.0)	54
4000 Hz	40.4 ± 12.7	15.6	53.0	Low (<34.5)	35
				High (≥34.5)	75

*Receiver operating characteristic (ROC) curves were used to determine cut-off values at each frequency.

### Associations of balance with sound component levels at 100 Hz, 1000 Hz and 4000 Hz after adjustment for confounders

We next determined the effects of sound component levels at 100 Hz, 1000 Hz and 4000 Hz on posturography scores in the logistic regression models adjusted for sex, BMI, smoking and alcohol consumption as confounding factors (Table 3). There was a significant association with track length of eyes open with sound levels at 100 Hz [high exposure group vs. low exposure group: odds ratio (OR) = 0.39, 95% confidence interval (CI) = 0.16–0.95, p = 0.039] (Table 3). Furthermore, the high exposure group at 100 Hz had smaller surface areas with both eyes open (OR = 0.26, 95% CI = 0.10–0.64, p = 0.004) and eyes closed (OR = 0.21, 95% CI = 0.08–0.53, p < 0.001) than those in the low exposure group (Table 3). However, we did not find any relationships between the scores of posturography and sound levels at 1000 or 4000 Hz (Table 3).

**Table 3:** Associations of balance with sound component levels at 100 Hz, 1000 Hz and 4000 Hz output from a PLD after adjustment for confounders (n = 110)^a^.

Frequencies	Exposure Groups^d^	Adjusted OR (95% CI) of balance^b^			
		Eyes open		Eyes closed	
		Track length (≥78.05 cm)^c^	Surface Area (≥2.78 cm^2^)^c^	Track length (≥85.00 cm)^c^	Surface Area (≥2.92 cm^2^)^c^
100 Hz	Low	Reference	Reference	Reference	Reference
	High	0.39 (0.16, 0.95)	0.26 (0.10, 0.64)	0.48 (0.20, 1.14)	0.21 (0.08, 0.53)
	*p*-Value	0.039	0.004	0.098	<0.001
1000 Hz	Low	Reference	Reference	Reference	Reference
	High	1.36 (0.59, 3.11)	1.05 (0.47, 2.38)	1.40 (0.62, 3.17)	1.03 (0.46, 2.29)
	*p*-Value	0.468	0.899	0.422	0.952
4000 Hz	Low	Reference	Reference	Reference	Reference
	High	1.32 (0.55, 3.16)	1.19 (0.50, 2.80)	1.11 (0.47, 2.64)	2.00 (0.84, 4.77)
	*p*-Value	0.536	0.696	0.812	0.116

^a^Logistic regression model was adjusted for sex, BMI, smoking status and alcohol intake per week as confounding factors.

^b^Abbreviations: OR, odds ratio; CI, confidence interval.

^c^Cut-off values of track length and surface area are median values.

^d^Cut-off values of sound levels (dB) at 100, 1000 and 4000 Hz output from a PLD to categorize subjects into two groups (low and high exposure groups) are shown in Table 2.

## Discussion

In this study, the group with high exposure (≥46.6 dB) at 100 Hz showed smaller values of track length and surface area in posturography, suggesting better balance, rather than worse balance, than in the group with low exposure at 100 Hz. Furthermore, in the logistic regression models adjusted for sex, BMI, smoking status and alcohol intake in which ORs of more than 1.00 mean worse balance, the group with high exposure (≥46.6 dB) at 100 Hz showed the significant ORs with less than 1.00. Thus, the results of this study showed a significant association of better balance with high exposure at 100 Hz compared to low exposure at 100 Hz. Our results partially correspond to results of a previous study showing that stimulation by white noise at 55 dB significantly improved balance in patients with imbalance^16^, although there was no mention in that report about which frequency was effective for balance. Sounds with frequencies below 100 Hz are defined as low frequency sound (LFS)^17^. Previous studies have shown that earphones connected to a PLD output sounds with a frequency range of LFS^18,19^. Thus, this pilot study showed for the first time that mild exposure to LFS included in music output from a PLD was associated with better balance in young adults.

In this study, the differences in sound levels at 100 Hz between cut-off values and background noise levels were about 5 dB. In a previous study, an increase of only 5 dB in a sound compared to the background noise level was shown to improve the recognition of the sound-source localization in humans^20^. Thus, it is possible that only a 5-dB difference of cut-offs at 100 Hz compared to the background noise levels has significant biological effects. On the other hand, there was no significant difference between hearing levels in the low and high exposure at 100, 1000 and 4000 Hz in this study (Fig. S1). All of the subjects were young and had exposure to sound components with levels below 85 dB at 100, 1000 and 4000 Hz. Thus, the sound levels were below the safety standard (85 dB) for 8 hours per day made by the International Standard Organization (ISO) and NIOSH^21^. Our previous study showed that acute exposure to low frequency noise 5 times at 100 Hz, 95 dB for 12 hours each time did not cause hearing loss in mice^22^. Thus, this study showed that the sound component with a frequency of at least 100 Hz in music from a PLD is associated with better balance, but not hearing loss, in young adults.

In this study, all postural stability parameters in female subjects were significantly better than those in male subjects, although the results of previous studies regarding the influence of gender on postural stability were inconsistent^23–25^. Smokers had significantly larger surface areas with both eyes open and eyes closed than those in non-smokers as shown in previous studies^26,27^. We then focused on the relative contribution of 100 Hz sound and further determined the relative contributions (%) of each confounder as well as the groups categorized by sound levels at 100 Hz in logistic regression models using Pseudo R^2^ values (Table S1). Sex had higher relative contributions to the track length of posturography, while smoking had higher relative contributions to the surface area of posturography. Moreover, the relative contributions of 100 Hz were the largest among other confounders to all scores of posturography in each logistic regression model. Notably, the relative contributions of 100 Hz to surface area with eyes closed were even more than 60%, which could present a good overall fit. Thus, our additional analysis suggests that the sound component at 100 Hz included in music is a greater contributor than others to postural stability of young adults in these models. In this study, we also determined the association of better balance with high exposure at 100 Hz compared to low exposure in the logistic regression models adjusted for listening time in addition to the confounders. The significance remained in the model with listening time in addition to the confounders (Table S2). Thus, the results suggest that the association of the sound component at 100 Hz with better balance in young adults is independent of listening time.

This study has several limitations. First, other sound exposure information (e.g., traffic noise and society noise) was not available in our study, and that might have had some potential confounding effects. Second, we chose to enroll young adults of the same age living in the same area in order to better control potential confounding. However, this strategy might limit the generalization of our results to people in other age groups. Further investigation of the effect of the sound component at 100 Hz on postural stability of people in other age groups is needed. Third, we did not measure sound components at frequencies other than the frequencies investigated in this study. Fourth, it was difficult to determine whether music preference influences the effect of the sound component at 100 Hz on balance in this study since most of the subjects chose several music categories (e.g., classic, pop and rock music) in the self-reported questionnaire. Further study is needed to determine the effect of the sound component at 100 Hz isolated from different music categories on balance. Finally, the mechanism underlying the association of better balance with mild stimulation of the sound component at 100 Hz in music is not clear. A previous study showed that galvanic vestibular stimulation (GVS) delivered as imperceptible white noise only at an intensity of 270 mA for healthy subjects or 450 mA for patients with bilateral vestibular dysfunction resulted in improvement of balance^28^. Our experimental studies have shown that constitutive exposure to sound with a peak of 100 Hz, 70 dB for 1 month caused impairment of the vestibule in mice^29,30^. Therefore, it is possible that the target organ for the sound component at 100 Hz in music is the vestibule.

In conclusion, our pilot study showed an association of the sound component at 100 Hz with more than 46.6 dB output from a PLD with better balance in young adults. This study suggests the importance of determining the influence of sound exposure with consideration of frequency on balance. Further studies are needed to determine the mechanism and the effects of stimulation of the sound component at 100 Hz on balance in humans.

## Materials and Methods

### Study subjects

This study was performed for 110 Japanese subjects with an average age of 20.4 ± 1.0 years (Table 1). The procedures were explained and informed consent was obtained from all of the subjects. None of the subjects had a history of ear disease and none of the subjects were suffering from illness at the time of the investigation. This investigation was performed using a self-reporting questionnaire on smoking, age, clinical history, weight and height. Body mass index (BMI) was obtained by using the following formula: weight in kg/height in meters^2^ (Table 1). This study was ethically approved by Nagoya University International Bioethics Committee (approval number 2013-0070) and Chubu University Ethical Committee (approval number 260019) following the regulations of the Japanese government.

### Measurement of postural stability

We measured postural stability with a gravicorder (GS-3000, Anima Co. Ltd., Japan). Each subject stood on the platform with eyes open for 1 min and then eyes closed for 1 min. Posturography parameters including track lengths and surface areas of the center of pressure were measured for analyses. Larger track lengths or surface areas indicate lower postural stability levels.

### Measurement of hearing levels

Hearing examinations at frequencies of 1, 4, 8 and 12 kHz were performed with duplicated measurements by pure tone audiometry (PTA) in a sound-proof room as described previously^31–35^. Sound signals at frequencies of 1, 4, 8 and 12 kHz were output to each subject until the thresholds of sound were identified. We measured hearing level at an extra-high frequency (12 kHz) because hearing level of this frequency is sensitive to environmental factors including smoking^32–36^. Hearing levels of the subjects were all measured by providing an initial stimulus of 5 dB followed by a stepwise increase in sound level by 5 dB.

### Measurement of sound levels output from a PLD

We used a sound level meter (TYPE 6236 with an FFT analyzer, ACO CO., LTD, Japan) in contact with an earphone of the portable music player, in order to perform triplicate measurements of sound levels of components at 100, 1000 and 4000 Hz in music and in the background. We asked the subjects to bring their portable music players without changing the sound levels to which they listened in their daily life.

### Statistical analysis

All statistical analyses were conducted using SPSS v24.0 (IBM Corp., Armonk, NY). The Mann-Whitney U test and Kruskal-Wallis H test were used for nonparametric data to determine a significant difference between two groups and among three groups, respectively, since the Shapiro–Wilk test showed that posturography parameters and hearing levels of participants were not normal distribution. We transferred categorized subjects into two or three groups according to sex (male and female), BMI (underweight, <18.5 kg/m^2^; normal range, 18.6–24.9 kg/m^2^; overweight, ≥25 kg/m^2^), current smoking status (no and yes) and tertiles of alcohol consumption (low, <5.25 mL/week; middle, 5.35–19.3 mL/week; high, ≥19.4 mL/week) and we compared the average track lengths and surface areas of the center of pressure with eyes open and eyes closed as well as hearing levels between two groups or among three groups. Then all participants were divided into two groups according to the cut-off values of sound intensity at each frequency output from the PLD obtained by receiver operating characteristic (ROC) curves and the highest Youden index^32^ in order to compare the postural stability levels between the two groups (Table 2). Finally, we performed multivariate analysis using a logistic regression model adjusted with sex, BMI, smoking status and alcohol consumption. In this model, we used dependent variables of two subgroups divided by median values based on posturography parameters and independent variables of two subgroups divided by the cut-off values of sound levels at 100, 1000 and 4000 Hz output from the PLD. Although previous studies showed that age could affect balance control^37^, all participants were young and the variance was only 1 year in this study. Hence, age was not considered as a confounding factor in this multivariate analysis. All tests were two-sided and significance threshold was defined as α = 0.05.

## Electronic supplementary material

Supplementary information
