# Supplementary material for: Improvement of balance in young adults by a sound component at 100 Hz in music
Source: Sci Rep. 2018 Nov 15;8:16894. doi: 10.1038/s41598-018-35244-3 (PMC6237978; doi:10.1038/s41598-018-35244-3)
Supplement: Supplementary file 1 — Supplementary information [file 41598_2018_35244_MOESM1_ESM.pdf]

Improvement of balance in young adults by a sound component at 100 Hz in music

Huadong Xu[1,3], Nobutaka Ohgami[1,2,3], Tingchao He[1,3], Kazunori Hashimoto[1,3], Akira Tazaki[1,3], Kyoko Ohgami[1], Kozue Takeda[2,3] and Masashi Kato[1,2,3,*]

Author Affiliation:

[1]Department of Occupational and Environmental Health, Nagoya University Graduate School of Medicine, Nagoya, Japan.

[2]Department of Biomedical Sciences, College of Life and Health Sciences, Chubu University, 1200 Matsumoto, Kasugai, Aichi 487-8501, Japan.

[3]Voluntary Body for International Health Care in Universities, Nagoya, Japan.

***Corresponding author:**

Masashi Kato M.D., Ph.D.

Department of Occupational and Environmental Health,

Nagoya University Graduate School of Medicine

Address: 65 Tsurumai-cho, Showa-ku, Nagoya, Aichi 466-8550, Japan.

Phone: +81-52-744-2122. Fax: +81-52-744-2124.

E-mail: katomasa@med.nagoya-u.ac.jp

Supplementary information

Figure legends

Figure S1. Associations of hearing levels with sound levels at different frequencies output from the PLD. Auditory thresholds (means ± SD) at 1, 4, 8 and 12 kHz in the low exposure group (solid square) and high exposure group (open circle) at 100 Hz (A), 1000 Hz (B) and 4000 Hz (C) of sound output from the PLD are presented. Cut-off values of sound levels at different frequencies are shown in Table 2. Significant differences ($^{*}p < 0.05$, $^{**}p < 0.01$) were analyzed by the Mann-Whitney *U* test.

Table S1. Relative contributions of variables to goodness-of-fit of each logistic regression model containing the five variables shown in the table based on Nagelkerke R square.

	Relative contribution (%)			
	**Eyes open**		**Eye closed**	
	**Track length** (≥ 78.05 cm)	**Surface Area** (≥ 2.78 cm^2)	**Track length** (≥ 85.00 cm)	**Surface Area** (≥ 2.92 cm^2)
**100 Hz**	31.01	55.19	27.59	72.22
**Sex**	29.11	6.01	24.14	1.11
**BMI**	13.29	6.56	19.83	11.11
**Smoking**	0.63	16.39	1.72	10.00
**Alcohol intake**	13.29	9.29	10.34	1.11
**Model redundancy**	25.95	6.56	16.38	4.44

Table S2. Associations of balance with sound component levels at 100 Hz, 1000 Hz and 4000 Hz in music after adjustment for listening time in addition to confounders (n=110)[a].

Frequencies	Exposure Groups[d]	Adjusted OR (95% CI) of balance[b]			
		Eyes open		Eyes closed	
		Track length (≥ 78.05 cm)[c]	Surface Area (≥ 2.78 cm^2)[c]	Track length (≥ 85.00 cm)[c]	Surface Area (≥ 2.92 cm^2)[c]
**100 Hz**	Low	Reference	Reference	Reference	Reference
	High	0.39 (0.16, 0.94)	0.25 (0.10, 0.63)	0.48 (0.20, 1.14)	0.21 (0.08, 0.54)
	p-Value	0.037	0.003	0.098	0.001
**1000 Hz**	Low	Reference	Reference	Reference	Reference
	High	2.29 (0.76, 6.97)	1.62 (0.56, 4.68)	2.46 (0.82, 7.44)	2.66 (0.89, 7.52)
	p-Value	0.143	0.374	0.110	0.101
**4000 Hz**	Low	Reference	Reference	Reference	Reference
	High	2.39 (0.55, 10.26)	0.93 (0.25, 3.45)	2.45 (0.57, 10.38)	2.55 (0.61, 10.67)
	p-Value	0.243	0.913	0.227	0.199

[a]The logistic regression model was adjusted for listening time, sex, BMI, smoking status and alcohol intake per week as confounding factors. For listening time, the subjects were categorized into three groups [short listening time group (< 10 min/day, n=15), middle listening time group (30-60 min/day, n=41) and long listening time group (≥ 60 min/day, n=54)] based on the self-reporting questionnaire.
[b]Abbreviations: OR, odds ratio; CI, confidence interval.
[c]Cut-off values of track length and surface area are median values.
[d]Cut-off values of sound levels (dB) at 100, 1000 and 4000 Hz output from a PLD to categorize subjects into two groups (low and high exposure groups) are shown in Table 2.

(A) 100 Hz

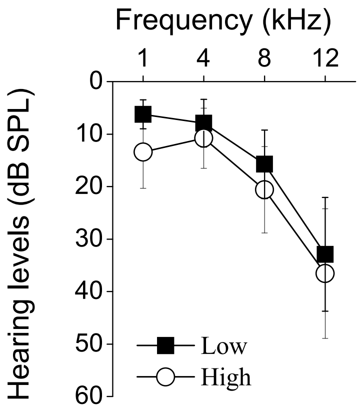

(B) 1000 Hz

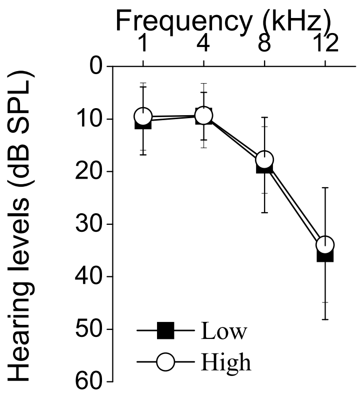

(C) 4000 Hz

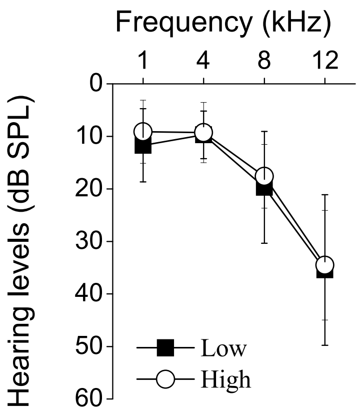

Fig. S1
